# Hyperactivation of p21-Activated Kinases in Human Cancer and Therapeutic Sensitivity

**DOI:** 10.3390/biomedicines11020462

**Published:** 2023-02-05

**Authors:** Deivendran Sankaran, Revikumar Amjesh, Aswathy Mary Paul, Bijesh George, Rajat Kala, Sunil Saini, Rakesh Kumar

**Affiliations:** 1 Signal Transduction and Molecular Pharmacology, The Institute of Cancer Research, London SW7 3RP, UK; 2 Centre for Integrative Omics Data Science, Yenepoya (Deemed to be) University, Mangalore 578018, India; 3 Cancer Research Program, Rajiv Gandhi Centre for Biotechnology, Thiruvananthapuram 695014, India; 4 Cancer Research Institute, Himalayan Institute of Medical Sciences, Swami Rama Himalayan University, Dehradun 248016, India; 5 Department of Medicine-Hematology and Oncology, Rutgers New Jersey Medical School, Newark, NJ 07103, USA; 6 Department of Human and Molecular Genetics, School of Medicine, Virginia Commonwealth University, Richmond, VA 23298, USA

**Keywords:** PAKs, cancer, metastasis, therapeutic sensitivity, combination therapy, clinical trials

## Abstract

Over the last three decades, p21-activated kinases (PAKs) have emerged as prominent intracellular nodular signaling molecules in cancer cells with a spectrum of cancer-promoting functions ranging from cell survival to anchorage-independent growth to cellular invasiveness. As PAK family members are widely overexpressed and/or hyperactivated in a variety of human tumors, over the years PAKs have also emerged as therapeutic targets, resulting in the development of clinically relevant PAK inhibitors. Over the last two decades, this has been a promising area of active investigation for several academic and pharmaceutical groups. Similar to other kinases, blocking the activity of one PAK family member leads to compensatory activity on the part of other family members. Because PAKs are also activated by stress-causing anticancer drugs, PAKs are components in the rewiring of survival pathways in the action of several therapeutic agents; in turn, they contribute to the development of therapeutic resistance. This, in turn, creates an opportunity to co-target the PAKs to achieve a superior anticancer cellular effect. Here we discuss the role of PAKs and their effector pathways in the modulation of cellular susceptibility to cancer therapeutic agents and therapeutic resistance.

## 1. Introduction

In general, the development and progression of human cancer to increasingly malignant phenotypes is polygenic in nature. The oncogenesis process is driven by the dysregulation of regulatory and feedback processes involved in the maintenance of overall cellular homeostasis, in terms of orderly regulation of proliferation, cell death, cellular motility, energy metabolism, inflammation, immune responses, gene expression, protein translation, epigenomic controls, etc. The dysregulation of cellular homeostasis during oncogenesis and metastasis occurs at multiple levels, either independently, concurrently, sequentially, or as a result of network effect(s). The underlying reason for such dysregulation varies from genomic alterations to extrinsic or intrinsic signals due to the dynamic nature of the tumor microenvironment, in addition to other regulatory variables [1,2,3,4,5].

Among various classes of regulatory molecules in cancer development and progression, kinases have been at the center of cancer therapeutics for about half a century. This line of investigation started with the discovery in the early 1980s that the encoded proteins of many transforming viruses are cell surface receptor tyrosine kinases and that such receptors mediate the effect of a variety of extracellular growth–stimulatory polypeptide signals. A large body of studies over the decades by both academic and pharmaceutical laboratories has firmly established a pivotal role for hyperactivation and genomic alterations in human epidermal growth factor receptors (HER1-4) in tumor progression; this consequently has made HERs the basis of most targeted therapeutics for the treatment of human cancer [6,7,8,9,10,11,12,13,14]. In addition to the dysregulation of receptor tyrosine kinases such as HERs, the process of oncogenesis is accompanied by hyperactivation and/or genomic and transcriptomic upregulation of intracellular serine/threonine kinases, such as p21-activated kinases (PAKs) [15,16,17,18]. Here we discuss recent advances in targeting PAKs in tumor cells and how the status of hyperactivated PAK-signaling components might modulate the susceptibility of cancer cells to anticancer drugs; this has emerged as one of the cellular determinants of therapeutic resistance. 

## 2. Founding Studies to Connect PAKs with Human Cancer

**Figure 1:**
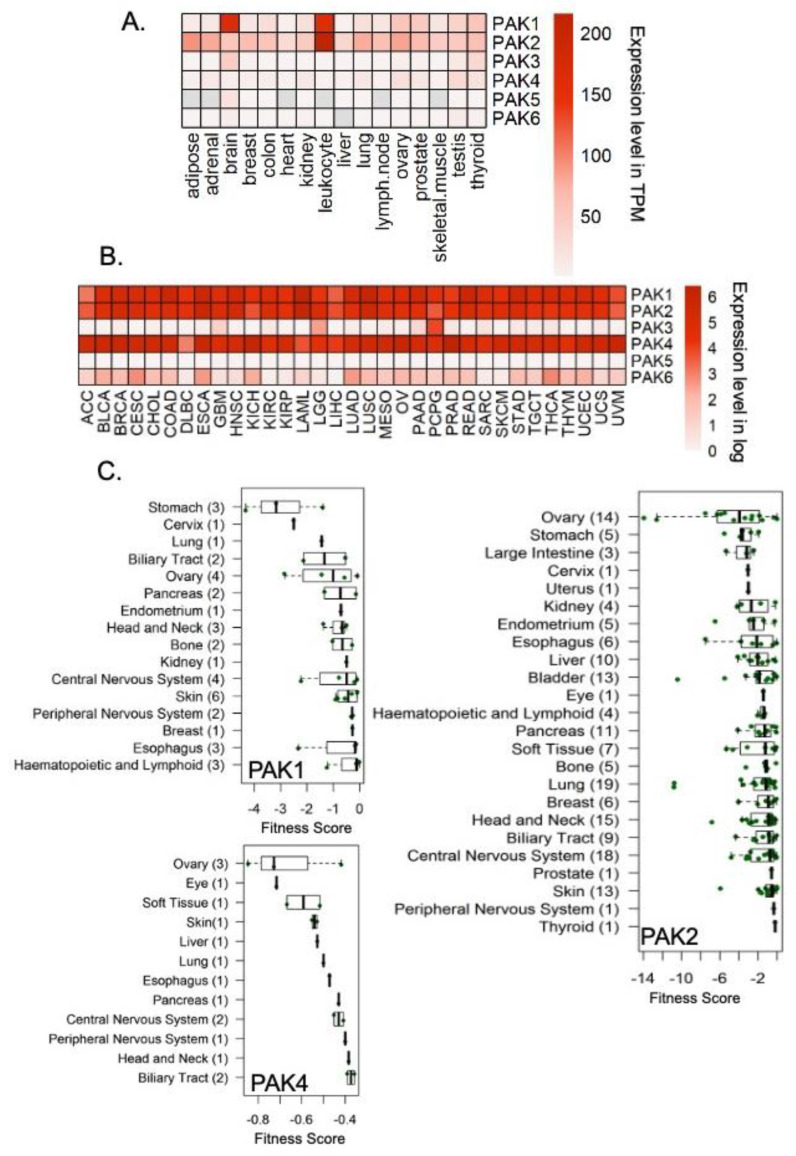
Expression of PAKs in normal tissues and human tissues. (**A**) Expression of PAK mRNAs in normal tissues using Illumina BodyMap data deposited in the EBI expression atlas database (E-MTAB-513); (**B**) Expression of PAK mRNAs in tumor tissue using TCGA dataset from GEPIA; (**C**) Status of PAK genes as fitness target genes with a significant dependency effect on the fitness of test cell lines upon knockdown of PAKs in different cancer cell lines in the Sanger Institute Cellular Dependency Dataset [27] Each dot, fitness value in one cell line; number in the bracket, number of cell lines.

**Figure 2:**
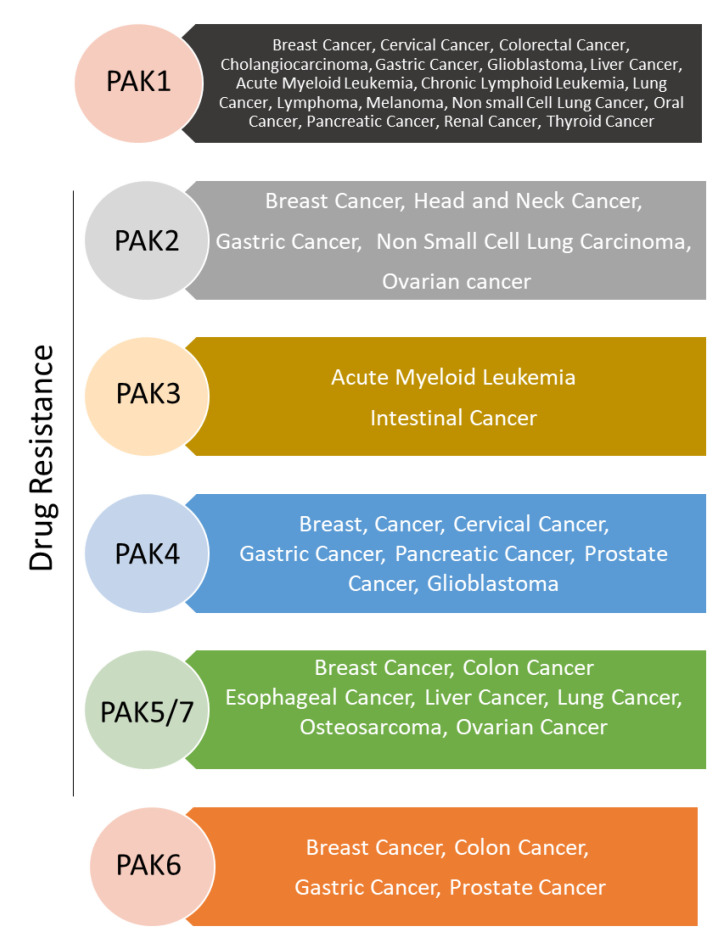
Diagram showing the drug resistance explored in different cancers by PAK.

**Figure 3:**
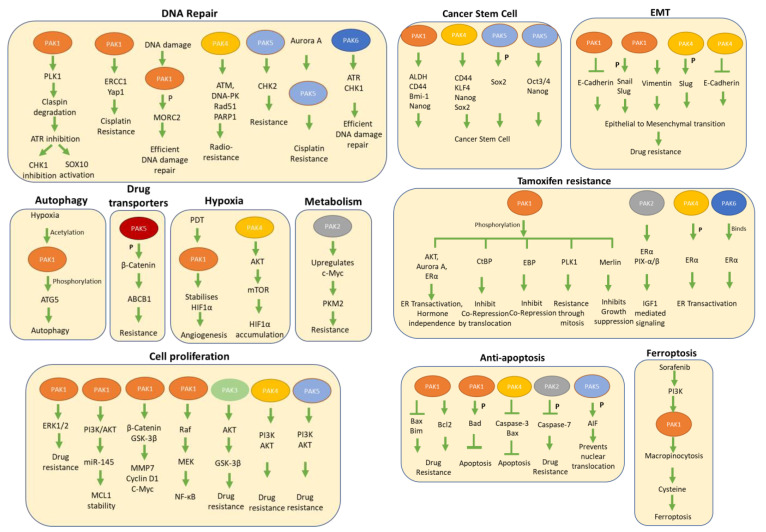
Diagram showing different pathways activated by PAK in regulating drug resistance.

The PAK family consists of six members, PAK1 through PAK6; PAK5 is also referred as PAK7 in some studies. PAK1-3 were initially characterized as effectors of the Cdc42 and Rac families of small GTPases and pursued for their role in directional motility in neural systems [15]. For the historical perspective and structural functional aspects of PAKs, readers are referred to previous comprehensive articles [15,16,17,18]. In an attempt to delineate the basis of the heregulin/neuregulin-β1 (HRG)-triggered invasion of HER2-overexpressing breast cancer cells in late 1996, PAK1 signaling was discovered to play a causative role in mediating the invasiveness of breast cancer cells [19]. At the same time, PAK1 was shown to be co-amplified with three other genes at chromosome 11q13.5 to 11q14.1 in breast cancer cells [20]. To further cement the role of PAK1 in HER-signaling, follow-up studies demonstrated a role of HER3 activation-linked HER2 phosphorylation in the rapid activation of PAK1 activity with a mechanism involving PI3-kinase recruitment to the HER2-HER3 heterodimers in HRG-stimulated breast cancer cells [19]. This first observation connected PAK1 signaling back to the cell surface activation of HER2 activation, leading to massive cytoskeleton remodeling and cellular invasiveness. Follow-up findings established that PAK1 hyperactivation and inactivation are sufficient to convert noninvasive breast cancer cells to invasive cells and vice-versa, respectively [21,22]. The next set of studies demonstrated the overexpression of PAK1 in human cancer specimens for the first time. PAK1 hyperactivation generally associates with polyploidy due to defective mitosis, and PAK1 interacts with the chromatin [22,23]. Similarly, murine models of PAK1 hyperactivation and inactivation in mammary glands were found to trigger hyperplasia and impair the differentiation of lobular structures through specific direct PAK1 substrates [24], further strengthening the role of the PAK family in breast cancer as well as other human cancers at large (Figure 1A,B). In addition, PAK1 activity and associate biological phenotypes could also be inhibited as a result of inhibiting the kinase activity of EGFR by a tyrosine kinase inhibitor ZR1839 in [25]. Hyperactivation of PAKs also supports cell survival over cell death [15], and the targeted depletion of PAK1, PAK2, or PAK4 in human cancer cell lines has been shown to compromise fitness and ability to survive [26,27] in a variety of cancer types (Figure 1C). Being a kinase, the functions of PAKs were expected to be mediated by the site-specific phosphorylation of their direct substrates, PAK-interacting proteins, PAKs’ sub-cellular redistribution, or PAK-regulated modulation of gene expression. The next phase of the field was devoted to discovering new direct PAK substrates and mechanistically implicating these in diverse cellular processes in cancer cells (Figure 2 and Figure 3). As expected, these and many other findings collectively propelled the notion that PAKs could be targeted in human cancer for therapeutic benefits, leading to the development of PAK-directed small molecules.

## 3. Activation Status of PAK Signaling and Therapeutic Sensitivity

As the PAK family members have been closely implicated in several aspects of cancer progression such as proliferation, improved cell survival, cell cycle progression, DNA damage response, invasion, and metastasis, etc. (Figure 2 and Figure 3), many of these processes also antagonize the effectiveness of anticancer therapeutics and thus are thought to be involved in the development of therapeutic resistance to commonly used anticancer drugs. In this context, it’s generally accepted that the cellular effects of cancer therapeutics over the course of treatment could range from the accumulation of acquired mutations to the rewiring of core survival pathways due to the compensatory effects of blocking or impairing the functionality of a given pathway [6,17,28]. To illustrate the role of PAKs in therapeutic resistance, here we provide the roles of PAK family members in different cancer types in the context of the activation of its downstream effector molecules and therapeutic sensitivity.

### 3.1. PAK1 Signaling and Therapeutic Resistance

Breast Cancer: Tamoxifen resistance is the major issue which develops during chemotherapy in patients with high expression of the estrogen receptor (ER)-α. PAK1 regulates tamoxifen resistance through phosphorylating ERα at Ser305 [24,29,30]. The phosphorylation of ERα by PAK1 results in conformational changes in the ER leading to phosphorylation at Ser118. Phosphorylation at Ser305 also leads to increased dimerization capacity and effective activation of downstream signaling by increased affinity to bind to the target promoters [31]. In addition, Ser305 phosphorylation by PAK1 induces Cyclin D1 expression [32]. PAK1 ameliorates tamoxifen resistance in breast cancer by phosphorylating other target substrates like Aurora A kinase [33], C-Terminal Binding Protein (CtBP) [9], and ErbB3-binding protein 1 (EBP1) [34]. PAK1 phosphorylates Aurora Kinase A at dual positions on Thr288 and at Ser342, accelerating the kinase activity by 100-fold [35]. Further, the transcriptional activity of ERα is increased through phosphorylation by Aurora A as well as AKT at Ser167 and Ser305 resulting in tamoxifen resistance [36]. In addition, Akt is activated by PAK1 phosphorylation at Thr308 and Ser473 [37]. PAK1 phosphorylates EBP1 at Thr261, leading to the inhibition of its co-repressor function [34,38,39]. Tamoxifen-resistant cells express high levels of Polo Like Kinase 1 (PLK1), and its phosphorylation at Ser49 by PAK1 induces its activity [40]. PAK1 by phosphorylating CtBP at Ser158 promotes tamoxifen resistance through a modulating co-repressor mechanism [9]. Corepressors use components of histone machinery to remodel the target chromatin for influencing the status of gene expression [41].

Upon DNA damage, PAK1 activity is stimulated via ATM-mediated phosphorylation of MORC2 at Ser739. MORC2 promotes the induction of H2A-γ. Phosphorylation-dependent activation of MORC2 regulates chromatin remodeling to facilitate efficient DNA damage repair [42]. Breast cancer treatment with Aurora kinase inhibitor (alisertib) and PAK inhibitor FRAX1036 shows promising results in luminal and HER2-enriched tumor subtypes [43]. In addition, cancer stem cells play a major role in developing drug resistance. Breast cancer stem cells enriched with cancer stem cell markers CD44 and ALDH promote chemoresistant mechanisms via Notch and Hedgehog pathways. In breast cancer, PAK1 associates with Janus-activated kinase (JAK2) to promote cancer stem population [44].

Non-Small Cell Lung Cancer (NSCLC): In NSCLC cells, irradiation increases the expression of PAK1. It also induces PAK1 phosphorylation at three residues (Tyr153, Tyr201, and Tyr285) and JAK2 phosphorylation. PAK1 is stabilized by JAK2 phosphorylation. PAK induces Snail phosphorylation at Ser246. The phospho-dependent activation of Snail promotes the EMT pathway through repression of E-cadherin and occludin [45]. Irradiation induces the expression of RAC1, which regulates the EMT markers vimentin and E-cadherin through the PAK1/LIMK1/Cofilin pathway [46]. NSCLC has higher expression of the protein kinase C iota (PKCι), and treatment with the PAK1 inhibitor (IPA-3) and the PKCi inhibitor (auranofin) is highly effective in EGFR and KRAS mutant NSCLC [47]. In NSCLC cells treated with cisplatin, PAK1 increases and promotes cisplatin resistance through MEK/ERK signaling. It promotes EMT signaling through phosphorylating GSK-3β at Ser9 and stabilizing it [48]. With H1975 cells, we demonstrated that DSG2 promotes osimertinib resistance [49].

Lung cancer: PAK1 provides resistance to tyrosine kinase inhibitors in both wild-type epidermal growth factor receptor (EGFR) and mutant cells [50]. PAK1 activity is determined by the subcellular localization of p120ctn [51]. Cytoplasmic p120ctn activated by ERK through PAK1 promotes drug resistance in lung cancer cells [51]. In lung cancer, the EGFR tyrosine kinase inhibitor osimertinib has been administered. Desmoglein-2 (DSG2) expression regulates resistance to osimertinib through PAK1. DSG2 interacts with EGFR in the cell membrane to stimulate EGFR signaling. EGF binds to Src through DSG2 and activates the EGFR-Src-Rac1-PAK1 signaling pathway. In lung cancer, PAK1 promotes inhibitors resistant to tyrosine kinase (Gefitinib and Erlotininb) in both wild-type EGFR and mutant cells. PAK1 upregulated in gemcitabine resistant cells is regulated by PI3K/AKT signaling. PAK1 modulates MCL1 phosphorylation at Ser159 via degradation of FBW7, thereby regulating the PI3K/AKT pathway [50].

Pancreatic cancer: In gemcitabine-resistant pancreatic cancer cells, Wang et al. [52] observed increased PAK1 expression and found that PAK1 activation induces resistance through modulating the Wnt/β-catenin signaling cascade. They also found that PAK1 inhibition sensitizes the cells toward all-trans retinoic acid (ATRA) treatment. In PDAC, MET receptor tyrosine kinase serves as a risk factor that is higher in chronic pancreatitis. Onartuzumab binds to the semaphorin domain of MET and prevents receptor activation, being administered along with other chemotherapy drugs. Growth factor–induced MET activates both PI3K, which activates through stathmin and PAK1 via Paxillin and promotes cancer progression. Treatment with onartuzumab along with PAK1 inhibitor or PAK1 inhibitor with PI3K inhibitor (Pictilisib) may result in a better prognosis [53].

Melanoma: Melanoma cells become resistant to many chemotherapeutic drugs alone or even in combination through upregulating antiapoptotic signaling and activating NF-κb signaling pathways. Treatment with cisplatin in melanoma induces RhoJ expression and in turn it activates PAK1 by phosphorylation at Ser199. PAK phosphorylation and RhoJ expression activates PLK1 and degrades Claspin through its phosphorylation. Due to the absence of Claspin, ATR cannot activate CHK1 and ATF2. It also induces Sox10 to promote cellular proliferation mediated through ATF2 [54]. In melanoma, BRAF mutant patients were treated with vemurafenib and PLX4720. PAK1 inhibition results in the sensitization of BRAF-mutant melanoma to a BRAF inhibitor. The combination of BRAFI and PAK1 inhibitors may serve as a better combination to overcome the resistant mechanism [55]. PAK1 is amplified and overexpressed in melanoma, and it is strongly associated with wild-type BRAF. PAK1 also phosphorylates both CRAF (Ser338) and MEK1 (Ser298) and activates MAPK signaling in BRAF WT cells. PAK1 inhibitor may have a prominent role in the treatment of melanoma with wild-type BRAF cells. PAK1 binds to MICAL1 and phosphorylates at Ser817 and Ser960. MICAL1 is a regulator of filamentous actin. MICAL1 phosphorylation results in F-actin disassembly. Activated PAK1 interacts with MICAL1 and regulates the binding with RAB7a and RAB10 to link Rho and Rab signaling [56]. Interestingly, Rab7a is known to increase drug resistance to cisplatin (CDDP) [57].

Distal cholangiocarcinoma: Distal cholangiocarcinoma (dCCA) is a highly aggressive cancer with poor prognosis. Patients treated with pemigatinib develop resistance toward it, and the mechanism was not well elucidated until Liu et al. [58] showed that Smad4 inhibits phosphorylation of β-catenin (Ser675) and intranuclear translocation. Protein phosphatase 1A (PP1A) dephosphorylates PAK1 (Thr423), which is responsible for the phosphorylation of β-catenin.

Decreased SMAD4 expression promotes pemigatinib resistance. Protein interaction studies confirmed that MYO18A interacts with PP1A and Smad4 proteins. Smad4 associates with Myo18A and PP1A to dephosphorylate PAK1, resulting in the inhibition of β-catenin to sensitize toward pemigatinib [58]. In cholangiocarcinoma cells, upon photodynamic therapy, increased PAK1 regulates the stability of HIF-1α by inhibiting HIF-1α ubiquitination–mediated degradation and promotes angiogenesis. Upon genotoxic stress induced by irradiation or etoposide treatment in the colon, glioblastoma and PDAC cells activated PAK1 phosphorylates CRAF at Ser338. Phosphorylated CRF interacts with CHK2, performs efficient DNA damage response, and promotes radio-resistance [59].

Glioblastoma: PAK1 is overexpressed in glioblastoma, and its higher expression results in poor prognosis [60,61]. PAK1 promotes cellular proliferation in glioblastoma through regulating the autophagy pathway during hypoxic conditions. Under hypoxic conditions, PAK1 is acetylated at K420 by Elongator complex protein 3 (ELP3). Acetylated PAK1 shows increased kinase activity by suppressing PAK1 dimerization. Subsequently, PAK1 phosphorylates ATG5 at Thr101 to stabilize ATG5. ATG5 phosphorylation also promotes the interaction of the ATG12-ATG5 complex with ATG16L1. Thus, PAK1 promotes autophagosome formation in hypoxic conditions and promotes cancer progression. This may be one of the mechanisms by which PAK1 could promote drug resistance in glioblastoma through hypoxia and autophagy signaling [62]. We also speculated that the pathway could function in colorectal, colon, and pancreatic cancers, as PAK1 is overexpressed in these cancers.

Liver cancer: In advanced hepatocellular carcinoma (HCC), sorafenib is currently administered as a standard therapy, and acquired resistance is inevitable in this process. Cancer cells induce macropinocytosis as a way to survive in amino acid–depleted condition to promote chemoresistance. In general, sorafenib treatment results in the depletion of cysteine, resulting in the “Ferroptosis” of cancer cells. A sorafenib-resistant population mediates micropinocytosis through modulating mitochondrial dysfunction and activates the PI3K and Rac/PAK1 pathway. Macropinocytosis reconstitutes the glutathione by replenishing cysteine and enables the cancer cells to thrive under drug treatment conditions [63].

Leukemia and lymphoma: In acute myeloid leukemia (AML), bone marrow stem cells (BMSCs) increase the expression of PAK1, resulting in the activation of ERK1/2 signaling to promote drug resistance through modulating the apoptotic pathway [64]. PAK1 is upregulated in chronic lymphocytic leukemia (CLL), and high levels of PAK1 are correlated with a poor prognosis. Ibrutinib is administered for CLL patients, and the patients tend to develop resistance toward ibrutinib as the treatment progresses. Wu et al., [64] through their high-throughput chromosome conformation capture (Hi-C) technology and multi-omic approach, discovered that PAK1 plays a pivotal role in resistance to ibrutinib. In CLL, PAK1 inhibitor IPA-3 inhibited cell proliferation, and its combination with ibrutinib resulted in a synergistic effect in ibrutinib-sensitive and -resistant cells, revealing its role in drug resistance [65].

In lymphoma, gene expression profiling in the drugs treated with BKM120, BEZ235, and BGT226 (PI3K and PI3K/MTOR inhibitors) in a panel of 60 cells showed higher expression of PAK1 association with PI3K resistance. PAK1 inhibitor treatment can restore the sensitivity of PI3K inhibition [66].

Other Cancers: PAK1 overexpressed in Oral cancers promotes cisplatin-mediated resistance through upregulating ERCC1 and YAP protein [67].

In renal cancer carcinoma (RCC) cells, silencing of PAK1 regulates stemness through affecting the phosphorylation levels of RAF1 (Ser338) and MEK1 (Ser298). Sunitinib induces the phosphorylation of PAK1 at Thr423 and promotes drug resistance through PAK1/NF-κB/IL-6 signaling in RCC cells [68].

In BRAF mutant thyroid cancers, treatment with PAK kinase inhibitor G-5555 reduces thyroid cancer cell progression. The combination of G-5555 and vemurafenib results in improved efficacy in BRAFV600E-mutated thyroid cancer cell lines [69].

A drug resistance mechanism is induced by certain miRNAs in several cancers. In cervical cancers, miR-509-5p is low in paclitaxel resistance cells. Through the biochemical approach, Xu et al. showed that miR-509-3p expression targets RAC1 signaling and affects the pathway, revealing a role of miRNA in drug resistance [70].

PAK1 also contributes to resistance to 5-fluorouracil stem cell markers Bmi1, Nanog, and CD44 in colorectal cancers [71]. In APC∆14/+ mice, depletion of PAK1 results in the increase of T-lymphocytes and B-Lymphocytes and suppresses intestinal tumor development, revealing its role in the immune response to tumors [72].

### 3.2. PAK2 Signaling in Therapeutic Resistance

Breast cancer: Increased IGF1R expression contributes to anti-estrogen resistance in breast cancer in patients with high expression of estrogen receptor. A kinome siRNA screen performed by Zhang et al. in 2018 identified PAK2 as one of the regulators of IGF1R-mediated antiestrogen. PAK2 is the strongest anti-estrogen resistance inducer. Knocking down PAK2 resulted in attenuation of IGF1R-mediated tamoxifen resistance [73]. It is also well known that PAK2 is higher in ER+ breast cancer patients and well-correlated with a poor prognosis. Zhang et al. [73], through their phospho-proteomic approach, have discovered that PAK2 plays a critical role in tamoxifen resistance by regulating IGF1R signaling with PAK-interacting exchange factors PIXα/β. HER2-positive breast cancer patients treated with lapatinib develop resistance to lapatinib as the treatment progress. PAK2 is identified as one of the targets and mediates drug resistance to lapatinib. Chang et al. [74] reported that PAK2 plays a pivotal role in developing lapatinib resistance through PAK2 inhibition on lapatinib-resistant cells. Increased PAK2 expression in breast cancer cells prevents apoptosis through regulating caspase-7. PAK2 inhibits apoptosis through binding with caspase-7 and phosphorylating caspase-7 at Ser30, Thr173, and Ser239 residues [75]. PAK2 regulates caspase-7 via two different mechanisms. Ser30 phosphorylation allosterically prevents its interaction with caspase-9, and phosphorylation at Ser239 makes caspase-7 inactive [76].

PAK2 promotes paclitaxel resistance in ovarian cancer mediated through p34/MLCK signaling [77].

Head and neck cancer: In head and neck cancer cells, PAK2 binds with c-Myc and stimulates c-Myc expression, resulting in high levels of PKM2 by binding to its promoter. Upregulation of PKM2 through PAK2 regulates the increased Warburg effect and promotes proliferation to mediate chemotherapeutic resistance. [78].

Gastric cancer: PAK2 lies in the downstream of Rho GDP dissociation inhibitor 2 (RhoGDI2) signaling. RhoGDI2 regulates the Rho family of GTPases and regulates the cancer progression and induces the chemoresistance in gastric cancer. [79]. Cho et al. (2012) elucidated the mechanism whereby PAK2 was regulated by RhoGDI2. Cathepsin D and PAK2 are regulated by RhoGDI2 in mediating the chemoresistance in gastric cancer cells [79,80].

NSCLC: Osimertinib (AZD9291) is being administered for patients harboring EGFR-mutation. Patients develop osimertinib during treatment. EGFR mutants activate the p21-activated kinase 2 (PAK2) which phosphorylates β-catenin to promote transcriptional activity, resulting in osimertinib resistance. ERBB3, by lying in the upstream, upregulates PAK2. Thus, β-catenin regulated by PAK2 and stimulated by ERBB3 drives osimertinib resistance in NSCLC patients [81].

### 3.3. PAK3 in Drug Resistance

In intestinal cancer, PAK3 promoted the resistance to radiotherapy mediated through AKT/GSK-3β signaling [82]. In AML patients who have undergone chemotherapy, expression of PAK3 is high [83].

### 3.4. PAK4 in Drug Resistance

Pancreatic cancer: PAK1 and PAK4 are known to be overexpressed in pancreatic cancer. Pancreatic cancer is often characterized by mutant KRAS mutation, and PAK4 is amplified in PDAC. Pancreatic cancer is treated by gemcitabine, and resistance to the drug is regulated by PAK4. Gemcitabine resistant cells express high levels of PAK4 and p-BAD. PAK4 inhibitor treatment along with KPT-9274 results in caspase-3 activation and increased expression of BAD and tumor-suppressive miRNAs [84]. Gene silencing of PAK4 results in the down-regulation of stem cell–contributing genes like Sox2, KLF4, Nanog, and Oct4, which is regulated by the phosphorylation of STAT3 at Tyr705 [85]. PAK4 also shows higher expression in PDAC stem cells with a CD24/CD44/EpCAM-positive population [85]. PAKib, a new PAK4 inhibitor, sensitizes PDAC cells and inhibits progression [86]. In pancreatic neuroendocrine tumors, everolimus is the FDA-approved clinical treatment. PAK4, upregulated in PNET, regulates anti-apoptotic signaling to modulate the resistance to everolimus. Treatment with the PAK inhibitor KPT-9274 along with KPT-9307 results in the decreased survival of the drug-resistant cells [87].

Other cancers: PAK4 on activation (phosphorylation at Ser474) is well correlated with prostate cancer progression. It stabilizes Slug through phosphorylation at Ser158 and Ser254, thereby inhibiting E-cadherin and promoting EMT signaling [88].

In cervical cancer cells: HIF-1α is stabilized by PAK4. During hypoxia, PAK4 activates 4EBP1 through mTOR signaling mediated via AKT to stabilize HIF-1α [89].

In gastric cancer cells: PAK4 provides resistance to cisplatin through the activation of the mitogen-activated protein kinase (MEK)/ERK and PI3K/Akt pathways [90].

In breast cancer cells: PAK4 phosphorylates ERα at Ser305 to promote tamoxifen resistance [91].

In glioblastoma cells, PAK4 is induced by irradiation and promotes radio-resistance through ATM and DNA -PK [92].

### 3.5. PAK5/7 in Drug Resistance

PAK7 in breast cancer regulates Wnt Signaling by binding to GSK-3β and β-catenin. PAK7 phosphorylates GSK-3β at Ser9 and inhibits β-catenin degradation, thereby promoting Wnt signaling [93]. It also regulates USP4 through the phosphorylation of the aspartyl aminopeptidase (DNPEP) enzyme. PAK5 phosphorylates DNPEP at Ser119 and regulates DNPEP through degradation. DNPEP negatively regulates USP4. USP4 has been implicated in drug resistance in glioblastoma [94]. Thus, PAK5 may regulate USP4 to promote drug resistance [95]. PAK5 phosphorylates apoptosis-inducing factor (AIF) in breast cancer and promotes breast cancer progression. PAK5 phosphorylates AIF at Thr281 and prevents the formation of a complex between AIF and importin α3 in breast cancer cells, resulting in the decreased nuclear translocation of AIF [96].

In colorectal cancers, PAK5 promotes migration and invasion through Cdc42 and integrin β1 [97].

PAK7 is often deregulated in gastrointestinal cancers and serves as an oncogene in colorectal, gastric, and esophageal cancer [98,99,100]. Resistance to cisplatin (CDDP) therapy remains a main concern in the treatment of esophageal cancer. He et al. found that Aurora A kinase is upregulated in cisplatin-resistant cells and inhibits apoptosis. They found that PAK7 is regulated by Aurora A, mediated through the transcription factor E2F1. Activated Aurora A stimulates the expression of PAK7 by increased binding of E2F1 onto the PAK7 promoter and promotes cisplatin resistance [101].

PAK5 resists apoptotic signals to promote chemoresistance induced by cisplatin in hepatocellular carcinoma via regulating CHK2 [102]. PAK5 induces the expression of ABCB1 (MDR1) to promote chemoresistance. PAK5 binds and phosphorylates β-catenin Ser675. PAK5 increases the expression of ABCB1 in resistant cells by binding onto the promoter. PAK5 regulates the expression of ABCB1 through phosphorylating β-catenin in liver cancer. Cancer cells upregulate the drug transporters as a mechanism to combat drug resistance. PAK5 and ABCB1 are positively correlated in HCC patient samples [103]. In HCC, cells expressing high PAK7 are resistant to radiation and enhance radiosensitivity through increasing H2AX [104]

In lung cancer cells, PAK5 interacts with Sox2 and phosphorylates Sox2. It also regulates the other stem cell markers such as ALDH, Nanog, Oct3/4, and Sox2 [105].

In osteosarcoma, cisplatin-resistant cells show higher expression of PAK5 and correlate with ezrin levels [106]

PAK5 overexpresses in ovarian cancers and is higher in patients who have undergone surgery followed by carboplatin and paclitaxel treatment. PAK5 regulates paclitaxel resistance [100]. It regulates the AKT (Ser473) and PI3K (PI3K p85 at Tyr458) pathways in ovarian cancer cells to regulate the EMT pathway [107].

### 3.6. PAK6 in Drug Resistance

PAK6 expression is higher in colon cancer patients undergoing treatment with 5-FU. Increased expression of PAK1 exploits the anti-apoptotic mechanism to promote resistance [108].

In gastric cancer, PAK6 modulates resistance to 5-fluorouracil/oxaliplatin and it is higher in patients treated with a combination of 5-fluorouracil and oxaliplatin [109]. PAK6 is overexpressed in oxaliplatin resistance cells and performs efficient DNA damage repair. PAK6 promotes resistance by homologous recombination in the nucleus by increasing Rad51 nuclear translocation to the damaged sites through ATR and CHK1 [110].

In prostate cancer, PAK6 phosphorylates BAD at Ser112 to mediate resistance to apoptosis induced by radiation. PAK6 silencing results in apoptosis, showing the importance of PAK6 in developing resistance [111].

In breast cancer, PAK6 interacts with the estrogen receptor to promote tamoxifen resistance [112].

## 4. Emerging PAK-Targeting Strategies

**Figure 4:**
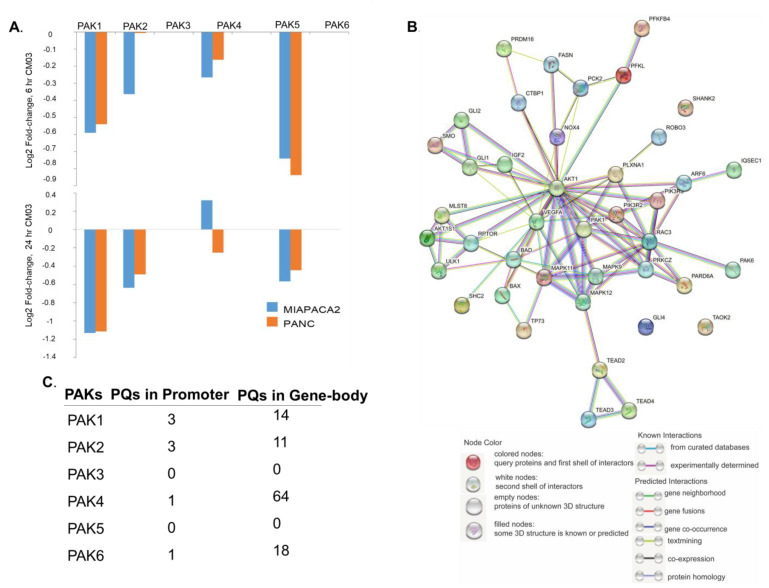
G4 ligands downregulate the expression of PAKs. (**A**) The levels of PAKs in CM03-treated pancreatic cancer cell lines previously reported in Marchetti et al. [113]; (**B**) Protein–protein interaction network among PAKs and other top downregulated genes in G4 ligand CM03−treated pancreatic cancer cells lines in Marchetti et al. [113] using STRING database [117]; (**C**) Number of G quadruplex in promoter regions within 1kb of TSS and the gene body of PAK family using the G4 mapping tool Quadebase2 [120].

While the notion of targeting PAKs by designer ATP-competitive and/or allosteric inhibitors is being actively pursued, recent studies aiming at stabilizing G-quadruplex structures (G4s, secondary DNA loop structures with four guanine strands) in human cancer cell lines by selective ligands revealed downregulation of several PAK family members as well as multiple components of the PAK pathway in human cancer cell lines [113]. Because G4 structures have been shown to overexpress in promoters and gene body regions of many cancer-associated genes, regulate gene transcription, and create genomic abnormalities (such as deletions, mutations, etc.) [114,115,116], this is a particularly exciting avenue for PAK-directed therapeutics. In this context, our analysis of RNA-sequencing data from the human pancreatic cancer cell lines PANC1 and MIAPACA2 treated with a designed G4 ligand CM03 revealed downregulation of PAK1, PAK2, PAK4, and PAK6 mRNAs (Figure 4A). Because Marchetti et al. also summarized a set of significantly downregulated genes [113], we hypothesized that many of these co-downregulated genes might be interacting with the PAKs. To this effect, using STING network tools [117], we found that, indeed, many of these co-downregulated genes are components of and interact with the PAK-pathways (Figure 4A). Interestingly, the noted downregulation of PAKs by CMO3 may not be restricted to the G4 ligand CMO3, because our analysis of published gene expression studies of cell lines treated with G4 ligands AQ1, 360A, and PehenDC3 [118,119] also found downregulation of PAK1 and PAK4. These observations raised a strong possibility of the prevalence of G4 structural motifs in the PAK genes. Using the G4 mapping tool Quadebase2 [120], our examination of the genomic structure of PAK genes suggested the presence of G4 structures in both the promoters and the gene bodies of the PAK genes (Figure 4C). The use of G4 ligands to target PAKs represents a promising new strategy to explore for impairing the activities associated with their genomic amplifications. It remains to determine if G4 ligands may be useful in reversing therapeutic resistance in settings of PAK-directed cancer therapeutics.

## 5. PAK-Directed Clinical Trials

Because of a widespread upregulation of several members of the PAK family in human cancer and its nodular role in mediating multiple transforming functions, a number of academic and pharmaceutical laboratories have developed PAK-directed inhibitory small molecules for targeting PAKs in cellular and animal models and, eventually, for taking promising candidate PAK-inhibitor(s) to cancer clinical trials (Supplementary Table S1) [121]. However, the early phase of such strategies was largely limited in scope due to drawbacks pertaining to target selectivity, safety, pharmacokinetics, and a tendency toward use as a single agent. Nevertheless, currently available PAK-inhibitors are proving extremely valuable tools to further delineate the mechanistic role of PAKs in oncogenic processes in cancer cell lines or animal models. For example, a clinical trial involving the first PAK4 inhibitor in solid tumors was terminated due to pharmacokinetics and safety issues [122]. Another clinical trial of an oral dual inhibitor of PAK4 and NAMPT in advanced solid tumors or non-Hodgkin’s lymphoma was terminated due to undisclosed reasons [123]. Recent studies suggest that nicotinamide phosphor-ribosyl-transferase (NAMPT)—a major rate-limiting enzyme and mandatory metabolic cofactor—is required for relapse of AML stem cells. Two ongoing, multicenter, promising trials of PAK4 inhibitors are testing the safety of the orally bioavailable dual inhibitors ATG-019 and KPT-9274, targeting the PAK4 and PAK4 and NAMPT in advanced solid tumors, in non-Hodgkin’s lymphoma [123], and in relapsed acute myeloid leukemia [124]. The authors expect that the results of these trials might serve as a guidepost for the clinical development of combinational targeted therapies involving PAK inhibitors.

## 6. Outlook

Since the discovery of the founding PAK1 in 1994, the PAK in the cancer biology field has made a tremendous advancement in basic and preclinical PAK research. A nodular role of PAKs in tumor biology has continued to be more apparent with each passing year. This, in turn, has contributed to the acceptance of the PAK family of kinases as a potential therapeutic target in many epithelial tumor types. Although for a variety of reasons the PAK inhibitors have not reached initial expectations as effective therapeutic agents in cancer patients, these inhibitors served as very useful research tools for gaining new mechanistic insights into the biology of PAK-family PAKs over the years. Clearly, the field needs a new strategy for developing improved molecules and delivery methods to achieve superior pharmacokinetics in cancer patients. Equally important would be the right selection of subsets of cancer patients with a known status of the expression or activity of PAKs as well as targets of other components of designer combination therapy and the preferred order for using such agents together. In addition, discerning the translational benefits of existing PAK-directed therapies in cancer patients remains a challenge due to a lack of validated biomarkers of an activated PAK pathway in patients with a given cancer type; thus, predicting the responsiveness versus resistance to anti-PAK agents also remains a challenge. Based on the lessons from HER-directed therapy, one way to move forward might be to use such validated biomarkers in selecting cancer patients on the basis of activated pathway(s) in the same patient for a given targeted combination therapy involving PAK inhibitors. This is just one of many new approaches that are being developed for significant anti-tumor benefits with accepted pharmacokinetics and toxicity for cancer patients.

## Data Availability

The data that support the findings of this study are available upon reasonable request (e.g., research purposes) from the authors.

## Supplementary Materials

The following supporting information can be downloaded at: https://www.mdpi.com/article/10.3390/biomedicines11020462/s1, Table S1: PAK inhibitors tested in clinical trials.
