# Supplementary material for: Hyperactivation of p21-Activated Kinases in Human Cancer and Therapeutic Sensitivity
Source: Biomedicines. 2023 Feb 5;11(2):462. doi: 10.3390/biomedicines11020462 (PMC9953343; doi:10.3390/biomedicines11020462)
Supplement: Supplementary file 1 [file biomedicines-11-00462-s001.zip › biomedicines-2176092-supplementary.pdf]

Hyperactivation of p21-activated Kinases in Human Cancer and Therapeutic Sensitivity

Deivendran Sankaran , Revikumar Amjesh, Aswathy Mary Paul, Bijesh George, Rajat Kala, Sunil Saini and Rakesh Kumar

Table S1: PAK inhibitors tested in clinical trials.

Trials	PAK	NCT Number	Title	Study Type	Study Phase	Study Design	Condition	Intervention	Status
1	PAK4	NCT00932126	Phase 1, Open Label, Dose-Escalation, Safety, Pharmacokinetic and Pharmacodynamic Study of Single Agent PF-03758309, an Oral PAK4 Inhibitor, in Patients with Advanced Solid Tumors	Interventional	Phase 1	Non-randomized	Advanced solid tumors	PF-03758309	Terminated
2	PAK4	NCT02702492	A Phase 1 Open-Label Study of the Safety, Tolerability and Efficacy of KPT-9274, a Dual Inhibitor of PAK4 and NAMPT, in Patients with Advanced Solid Malignancies or Non-Hodgkin's Lymphoma	Interventional	Phase1	Non-randomized	Solid tumors, non-Hodgkin's lymphoma	KPT-9274<br>Niacin ER<br>Nivolumab	Terminated
3	PAK4	NCT04281420	A Phase I Open-Label Study of the Safety and Tolerability of ATG-019, a Dual Inhibitor of PAK4 and NAMPT, in Patients With Advanced Solid Tumors or Non-Hodgkin's Lymphoma	Interventional	Phase 1	Non-randomized	Solid tumors, non-Hodgkin's lymphoma	ATG-019<br>ATG-019 + Niacin ER	Ongoing
4	PAK4	NCT04914845	A Phase 1 Open-label Study of KPT-9274 in Patients With Relapsed and Refractory Acute Myeloid Leukemia	Interventional	Phase 1	Non-randomized	Acute Myeloid Leukemia (relapsed, refractory)	KPT-9274	Ongoing
